# The morphometric of lycopsid sporophylls and the evaluation of their dispersal potential: an example from the Upper Devonian of Zhejiang Province, China

**DOI:** 10.1186/s12862-021-01933-3

**Published:** 2021-11-03

**Authors:** Yi Zhou, De-Ming Wang, Le Liu, Pu Huang

**Affiliations:** 1 Key Laboratory of Orogenic Belts and Crustal Evolution, School of Earth and Space Sciences, Peking University, Beijing, 100871 China; 2 College of Geoscience and Surveying Engineering, China University of Mining and Technology (Beijing), Beijing, 100083 China; 3 Nanjing Institute of Geology and Palaeontology, Chinese Academy of Sciences, Nanjing, 210008 China

**Keywords:** Lycopsids, Late Devonian, Dispersal, Reynolds number

## Abstract

**Background:**

Previous studies have discussed the special structural adaptations of Late Palaeozoic lycopsids, for example, the dispersal potential of reproductive organs. Based on materials from the Upper Devonian Wutong Formation in Changxing County, Zhejiang Province, China, we now analyze the morphometric and perform some calculation to evaluate the dispersal of sporophyll units of lycopsids.

**Results:**

The fossil sporophyll units are divided into two types in view of obvious difference in shape and we name two new (form) species for them. We also analyze the falling process and give the calculation method of dispersal distance.

**Conclusions:**

The fossil sporophyll units show relatively poor potential of wind dispersal compared with modern samaras, and show potential adaptation to the turbulent environment.

**Supplementary Information:**

The online version contains supplementary material available at 10.1186/s12862-021-01933-3.

## Background

Lycopsids are regard as one of the earliest lineages of vascular plants [1]. They evolved arborescent habit in the Mid-Late Devonian when the forests appeared [2, 3]. Arborescent lycopsids continued to increase in size and became the most conspicuous components of Carboniferous swamp forests [4, 5]. They displayed three different megaspore dispersal mechanisms, and one type is represented by *Lepidophloios*, in which the megasporangium-sporophyll units (named lepidocarps) are disseminated [6, 7]. This type of megasporophyll units was discussed in relation to function, and the sporophyll lamina like a wing may represent an adaptation for wind dispersal [8]. Phillips has posited that such sporophyll lamina acts in part as a sail and the dispersal of lepidocarps may proceed through the water [6]. Habgood constructed the models of lepidocarps to imitate the fossil sporophylls and performed a series of experiments to explore the functions of sporophylls in the process of flotation and fall [9]. As the results show, the models’ high terminal velocity of fall and the likely orientation suggest a poor flotation properties in water. On the other hand, the models spin stably and slow down the increase of falling rate, making more time for dispersal when they fall in the air. This, coupled with considerable height of mature *Lepidophloios*, would make the wind dispersal a viable mean.

**Fig. 1:**
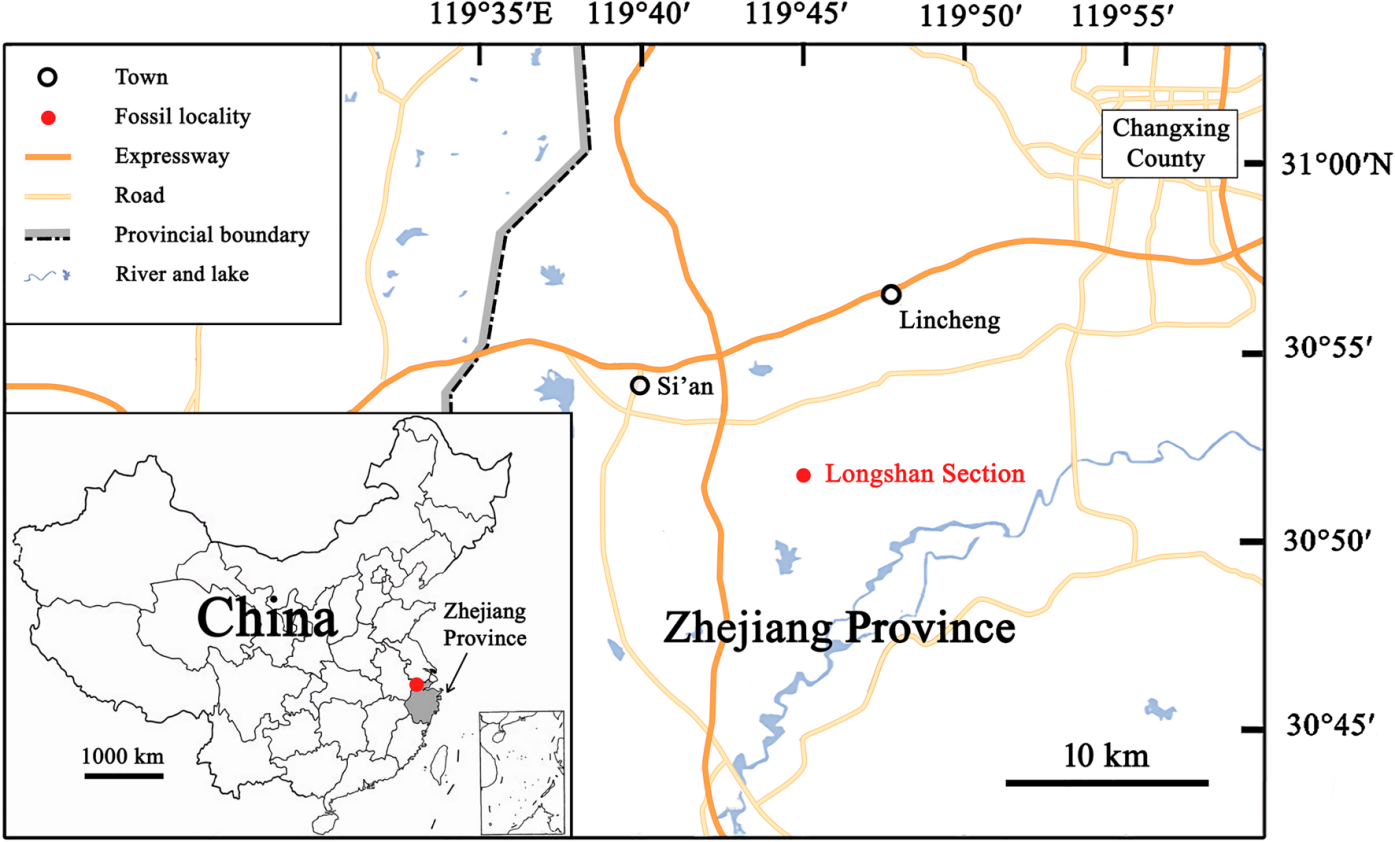
Map showing the location (Longshan Section) of the fossils

**Fig. 2:**
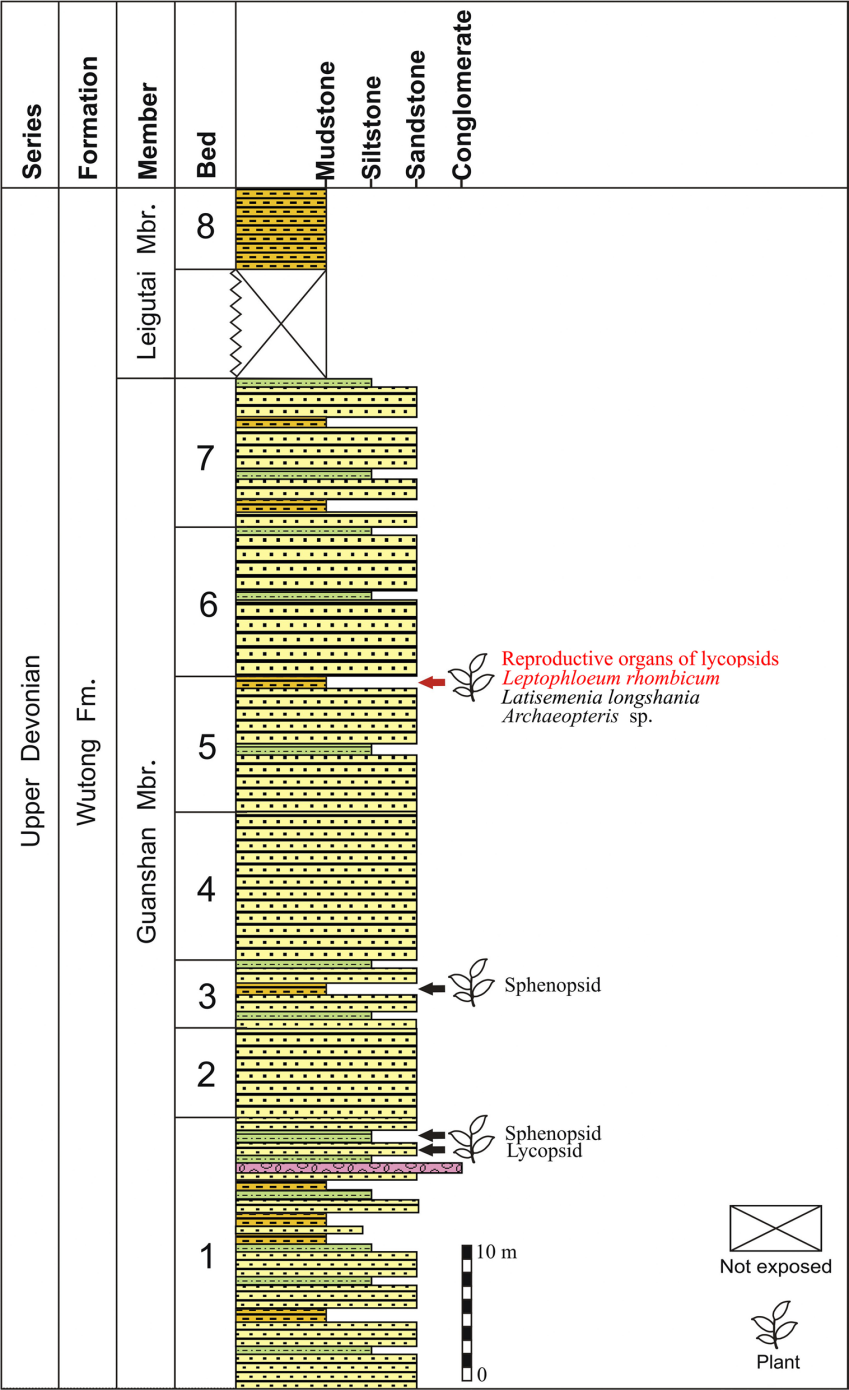
Stratigraphic column at Longshan section, Changxing County (Zhejiang, China), showing the lithology and sequence of strata, and the beds of the fossil plants ( modified from Fig. S3 of Wang et al. 2015 [20])

However, the wind conditions in the habitat of the lycopsids also need to be considered and the quantitative result may be different to some extent. Unlike the modern plant taxa, it’s impossible to observe the shedding of reproduction parts of the extinct taxa in the field or experiment. In this article, we describe the reproductive organs of the lycopsids from the Upper Devonian of Zhejiang Province, China (Figs. 1 and 2). In one of the Habgood’ s experiments, models of lepidocarps were released in still air at a height of 9 m, and the terminal velocities were recorded [9]. Based on the above experimental data, we use mathematical and physical methods to evaluate the ability of the sporophyll units for wind dispersal. We compare their characters with modern samaras, and discuss their adaptation to the environment.

## Results

### Reproductive organs

**Fig. 3:**
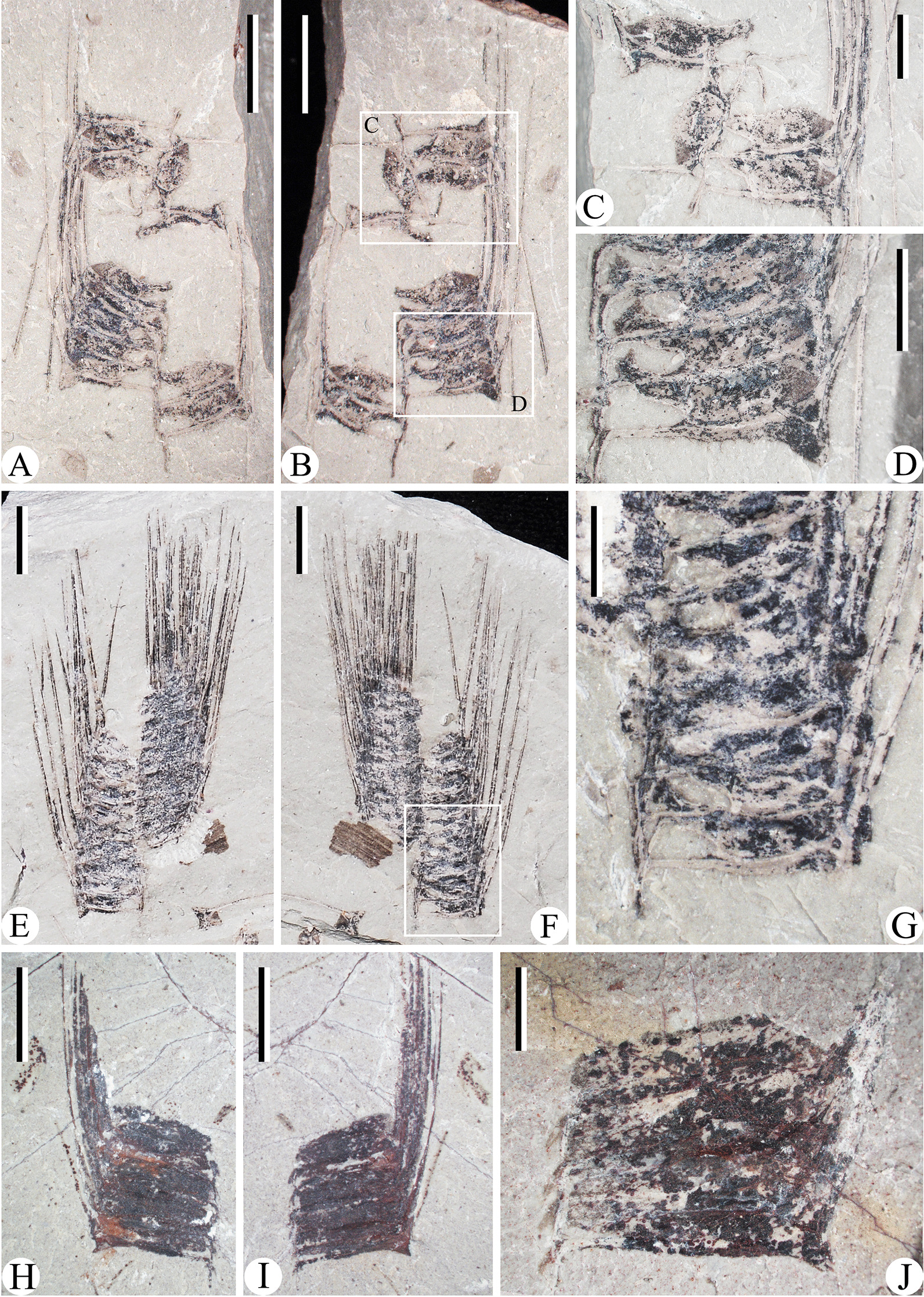
The “small” strobili **A**–**G** and the “large” strobili (**H**–**J**). **A**, **B** Part and counterpart of a “small” strobilus, PKUB15005a, b. **C** Enlargement of B (upper quadrangle), shows the sporophylls. **D** Enlargement of B (lower quadrangle), the adaxial part of each sporangium curl upwards. **E**, **F** Part and counterpart of well-preserved “small” strobilus, PKUB15007a, b. **G** Enlargement of F (quadrangle). **H**, **I** Part and counterpart of a “large” strobilus, PKUB15006a, b. **J** A fragment of “large” strobilus. PKUB15029. *Scale bars* 5 mm (**A**, **B**, **E**, **F**, **H**, **I**), 2 mm (**C**, **D**, **G**, **J**)

A sporophyll shows a distal lamina (an expanded blade-like portion) and a pedicel (a narrow stalk-like proximal portion). In lateral view, the lamina appears linear in shape, turns upwards at an angle of 70°–85° from the pedicel and then slightly curves adaxially (Fig. 3). The lengths of sporophyll pedicels are concentrated at 3.4–4.8 mm and 6.0–9.8 mm. The sporophylls with short pedicel length (3.4–4.8 mm) mostly have developed heels, distinctive serrate margins and bear spherical to ellipsoidal shaped sporangia. They are named the “small” sporophylls. In the other hand, the sporophylls with long pedicel length (6.0–9.8 mm) have less obvious heels, delicate enations and elongated sporangia. They are accordingly named the “large” sporophylls.

The strobili with sporophylls are fragmentary (Fig. 3). The strobili bearing “small” sporophylls tend to be narrow (Fig. 3A–G). One of such strobili is about 31 mm long and 10–12 mm wide, and the sporophylls are inserted at about 90° onto the strobilar axis (Fig. 3E–G). Each sporophyll bears one adaxial sporangium, 2.6–4.8 mm long and 1.5–1.8 mm high. The adaxial parts of sporangia are apparently separated from the sporophyll pedicels. In the strobili with “large” sporophylls, the sporangia adhere to pedicels even in the most basal parts (Fig. 3H–J). The diameters of such sporangia range from 6.9 mm to 9.2 mm and the heights are 1.8–2.6 mm.

**Fig. 4:**
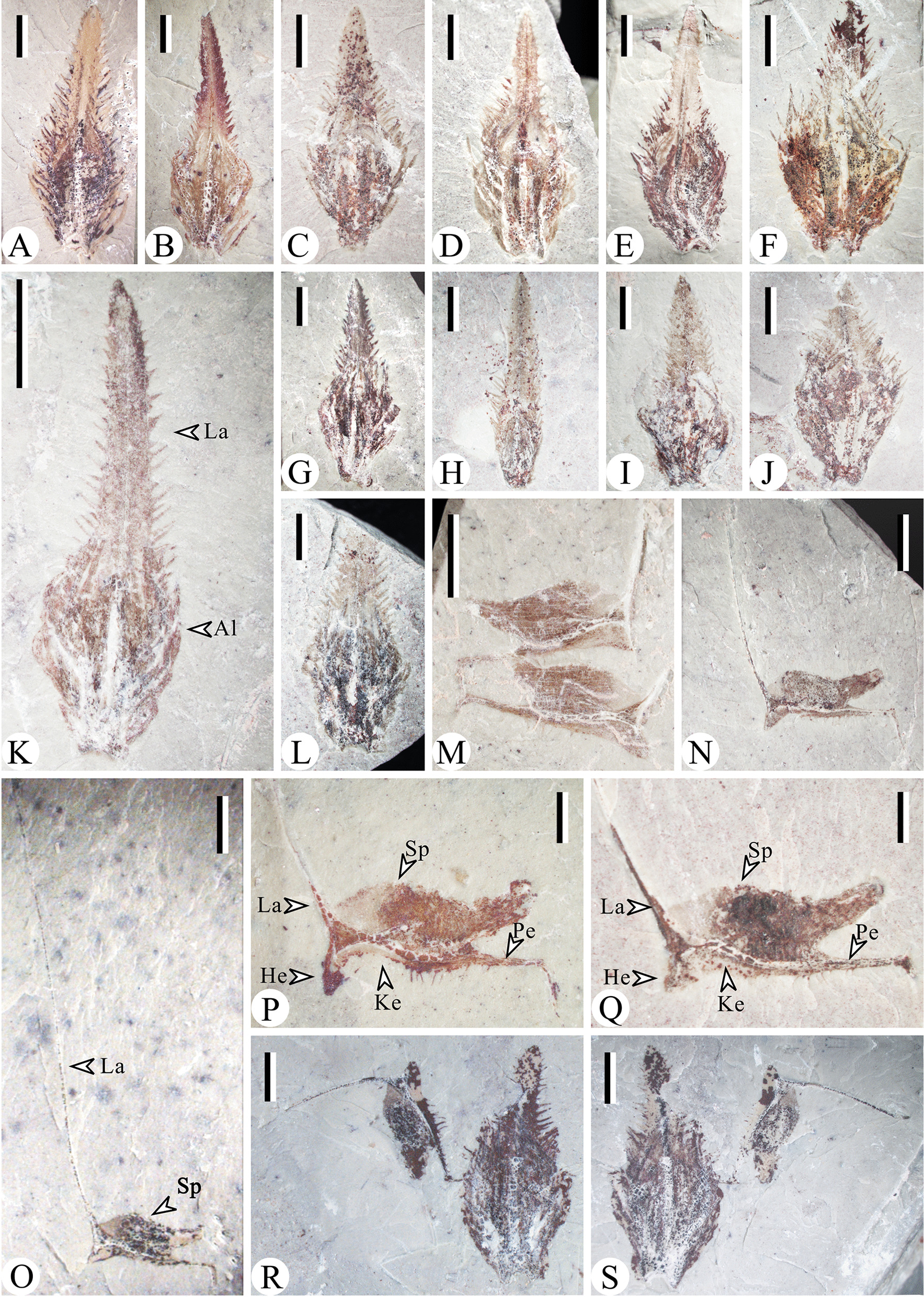
The “small” Sporophylls (Al, Alations; He, heel; Ke, keel; La, lamina; Pe, pedicel; Sp, sporangium). **A**– Abaxial views of sporophylls, PKUB15080, 15082, 15077, 15062, 15074, 15066, 15072, 15069, 15008, 15063, 15090, 15089. **M**–**Q** Lateral views of sporophylls. PKUB15034, 15016, 15101, 15102, 15001. Q shows he slender laminar. (R-S) Part and counterpart of sporophylls. PKUB15075a, b. *Scar bars* 2 mm

**Fig.5:**
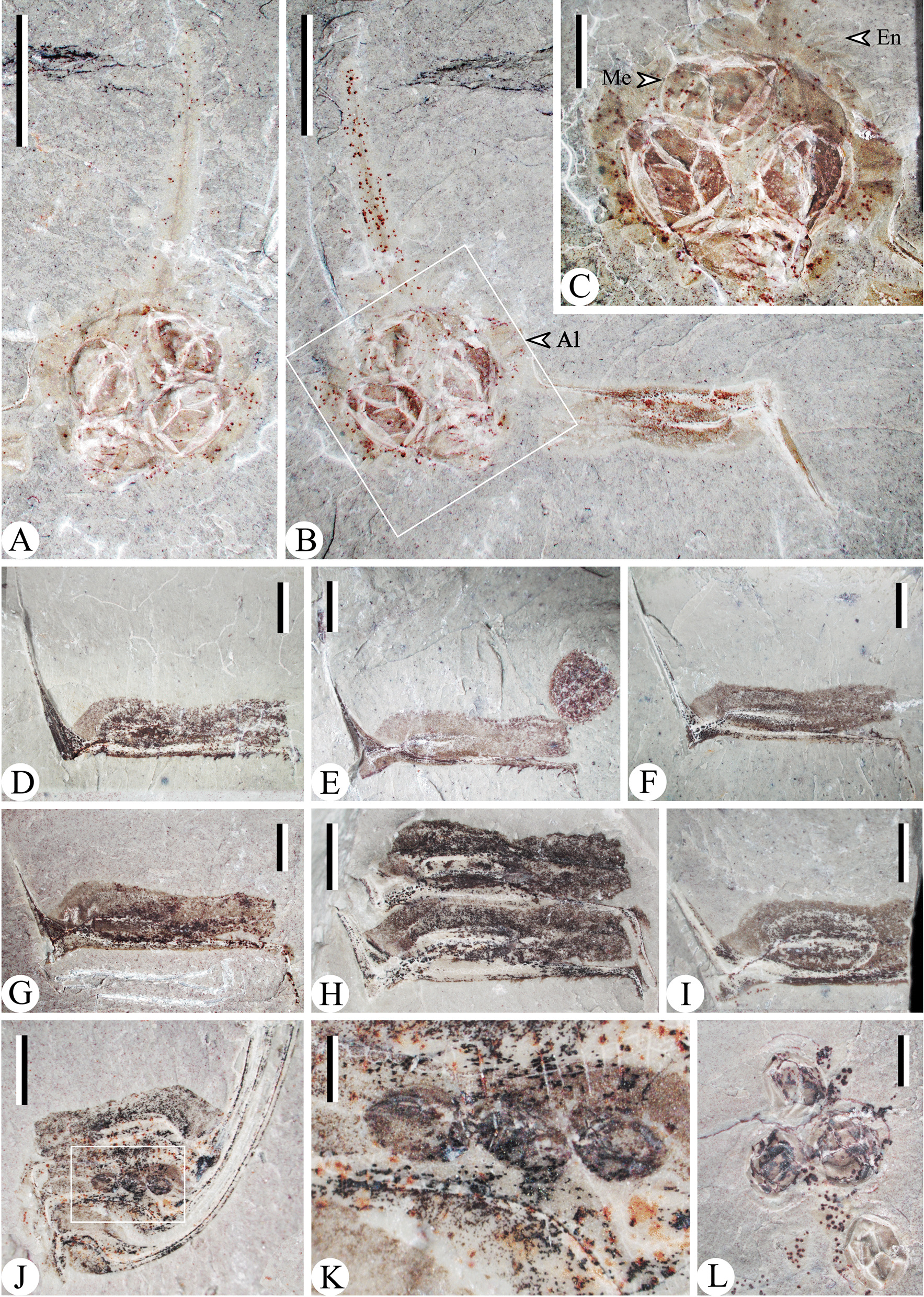
The “large” Sporophylls (*Al* alations, *En* enations, *Me* megaspore). **A**, **B** Lateral views and adaxial views of sporophylls. PKUB15068a, b. **C** Enlargement of B (quadrangle), showing 4 circular megaspores in a sporangia and the prickle in the margin of the sporophylls. **D**–**I** Lateral view of sporophylls. PKUB15053, PKUB15022, 15020, 15028, 15100, 15061. **J** A fragment of “large” strobilus. PKUB15012. **K** Enlargement of J (quadrangle), shows three megaspores. **L** Dispersed trilete megaspores. PKUB15071. *Scar bars* 5 mm (**A**, **B**), 2 mm (**C**, **D**, **E**, **F**, **G**, **H**, **I**, **J**), 1 mm (**K**).

The “small” dispersed sporophylls have laminae with serrate margins (Fig. 4A–S), while the “large” dispersed sporophylls also have delicate enations (Fig. 5A, B). The alations (the horizontal extension of pedicels) are wider than or equal to the sporangia in both two kinds of sporophylls. The heels of “small” sporophylls evidently decurrent and extend to the keels (Fig. 4M–S), and in “large” sporophylls the heels are relatively unobvious (Fig. 5D–I).. Some megaspores were preserved in the specimens of “large” dispersed sporophylls (Fig. 5A–L). The elongated sporangia are discoid in front view, 6.8–9.4 mm in diameter and 1.8–2.8 mm in height. Four equally sized megaspores (diameters ranging from 2.6–3.4 mm) occur within a sporangium of a “large” individual sporophyll (Fig. 5A–C) or occur in a cluster (Fig. 5L). The trilete rays are present in all these megaspores. Examining a sporangium of a strobilus, three megaspores (diameters range from 1.4–1.7 mm) are seen in the lateral view and the fourth one may be covered (Fig. 5J–K).

### Morphometrics of sporophyll units

**Fig. 6:**
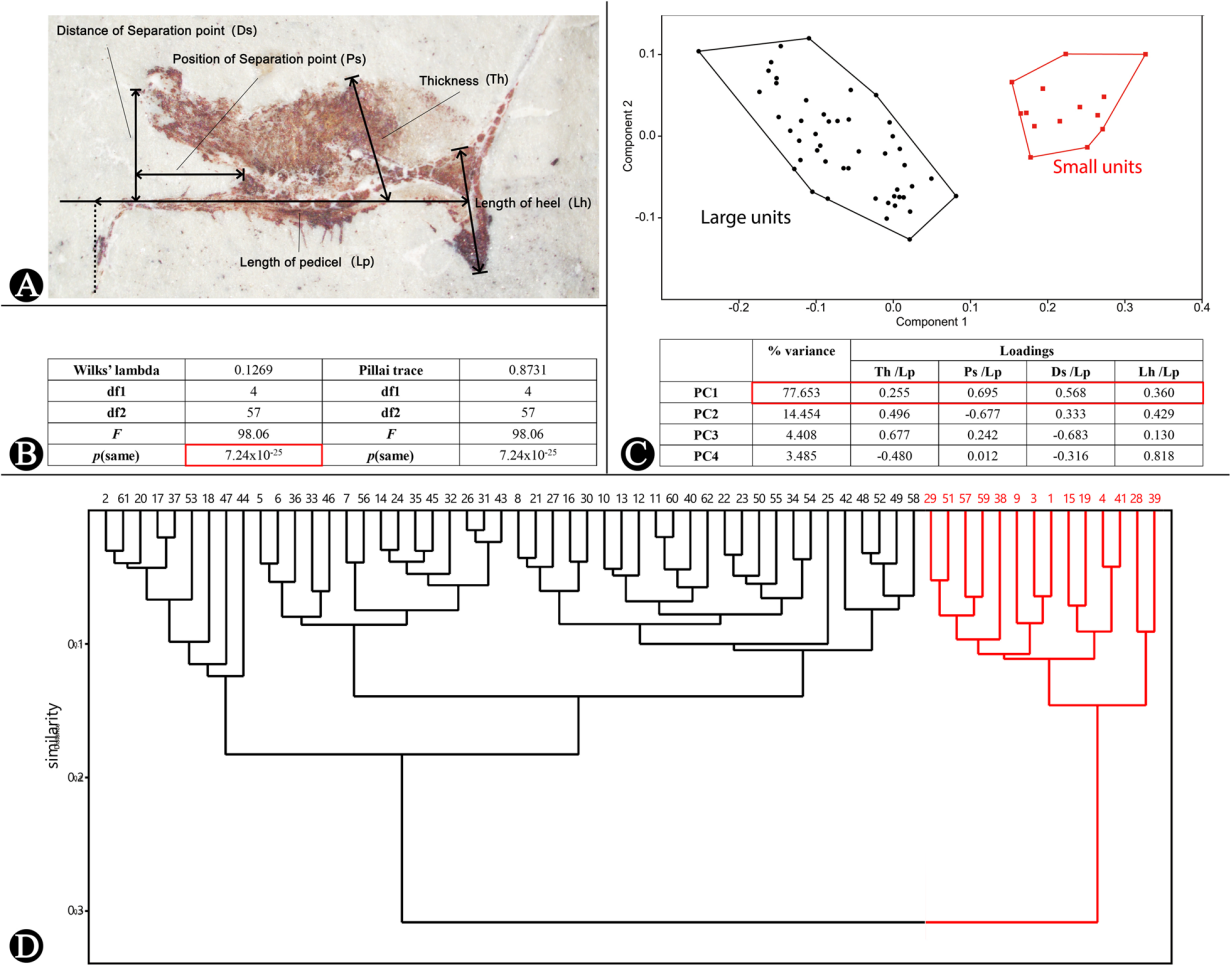
The measurement of sporophylls and analysis of measured data. **A** The measurement of sporophylls. *Lp* length of pedicel, *Th* thickness of sporangia, *Lh* length of heel, *Ps* position of separation, *Ds* distance of separation. **B** The result of MANONA (Multivariate analysis of variance). The p (same) value means the probability of the hypothesis that “small” sporophyll units and “large” sporophyll units are same in shape. In this analysis, $p (\mathrm{same})=7.24\times {10}^{-25}$, which means that the difference in shape is for certain. **C** The Principal Component Analysis of the measured data. Red Square, “small” sporophyll units; Black Dot, “large” sporophyll units. The form at the bottom shows the variance and loadings. **D** The Hierarchical clustering (UPGMA) of measured data (the similarity metric is Euclidean). Red numbers, “small” sporophyll units; Black numbers, “large” sporophyll units.

We choose several indicators to describe the shape of sporophylls (Fig. 6A). The lengths of sporophyll pedicels are used to represent the “sizes”. To guarantee the independence of features, we also use the proportions of the other indicators to the pedicels’ lengths in order to describe the “shapes” (Additional file 1: Table S1). Additional file 1: Table S1 includes the measurements of 62 sporophylls which constitute the morphological database. We perform the Multivariate analysis of variance (Fig. 6B), Principal Component Analysis (Fig. 6C) and Cluster Analysis (Fig. 6D) of this database. As the analysis results show, “small” sporophyll units take up quite different morphological scale space from “large” sporophyll units (Fig. 6B, C). The relative character loadings show that each character makes significant contribution to the separation. It means the shapes of the “small” sporophylls are markedly different from the “large” sporophylls, and we tend to think two kinds of sporophylls are not the different development stages of one sporophyll. Finally, we name two new (form) species for them.

**Systematics**

**Class Lycopsida**

**Order Lepidodendrales**

Family Incertae sedis

Genus *Lepidophylloides* Snigirevskaya, 1958

*Type species*: *Lepidophylloides aciculum* (Reed) Snigirevskaya, 1958

***Lepidophylloides longshanensis Zhou et al.*** (The “small” sporophyll units)

Diagnosis: A sporophyll consists of a distal lamina and a pedicel, with serrate margin. The alations are wider than or equal to the sporangia. The heels are evidently decurrent and extend to the keels. Sporangia are on the adaxial side of sporophylls, and the adaxial parts of sporangia are apparently separated from the sporophyll pedicels. Pedicels are 3.4–4.8 mm long, alations are 3.4–5.3 mm wide, heels are 0.9–1.5 mm high, and laminae are12-20 mm long. Sporangia are 2.6–4.8 mm long and 1.5–1.8 mm high.

Holotype: PKUB15075a, b. (Fig. 4R, S).

Paratype: PKUB15062, 15074, 15089, 15101, 15102. (Fig. 4D, E, K, O, P).

Etymology: Specific epithet indicates that the plant was collected in Longshan section.

Locality and horizon: Longshan, Lincheng, Changxing, Zhejiang, China; Guanshan Member of Wutong Formation; Upper Devonian (Famennian).

***Lepidophylloides changxingensis Zhou et al.*** (The “large” sporophyll units).

Diagnosis: A sporophyll consists of a distal lamina and a pedicel, with delicate enations. The alations are wider than or equal to the sporangia. The heels are unobvious. Horizontally elongated megasporangia are on the adaxial side of sporophylls. Four equally sized megaspores occur within a sporangium. Megaspores bear trilete rays. Pedicels are 6.0–9.8 mm long, alations are 6.9–9.8 mm wide, heels are 0.5–2.0 mm high, and lamina are 15–26 mm long. Megasporangia are 6.8–9.4 mm long and 1.8–2.8 mm high. Megaspores are1.4–3.4 mm in diameter.

Holotype: PKUB15068a, b. (Fig. 5A, B).

Paratype: PKUB15022, 15028 (Fig. 5D, G).

Etymology: From Changxing county where the plant was collected.

Locality and horizon: Longshan, Lincheng, Changxing, Zhejiang, China; Guanshan Member of Wutong Formation; Upper Devonian (Famennian).

### Falling model

**Fig. 7:**
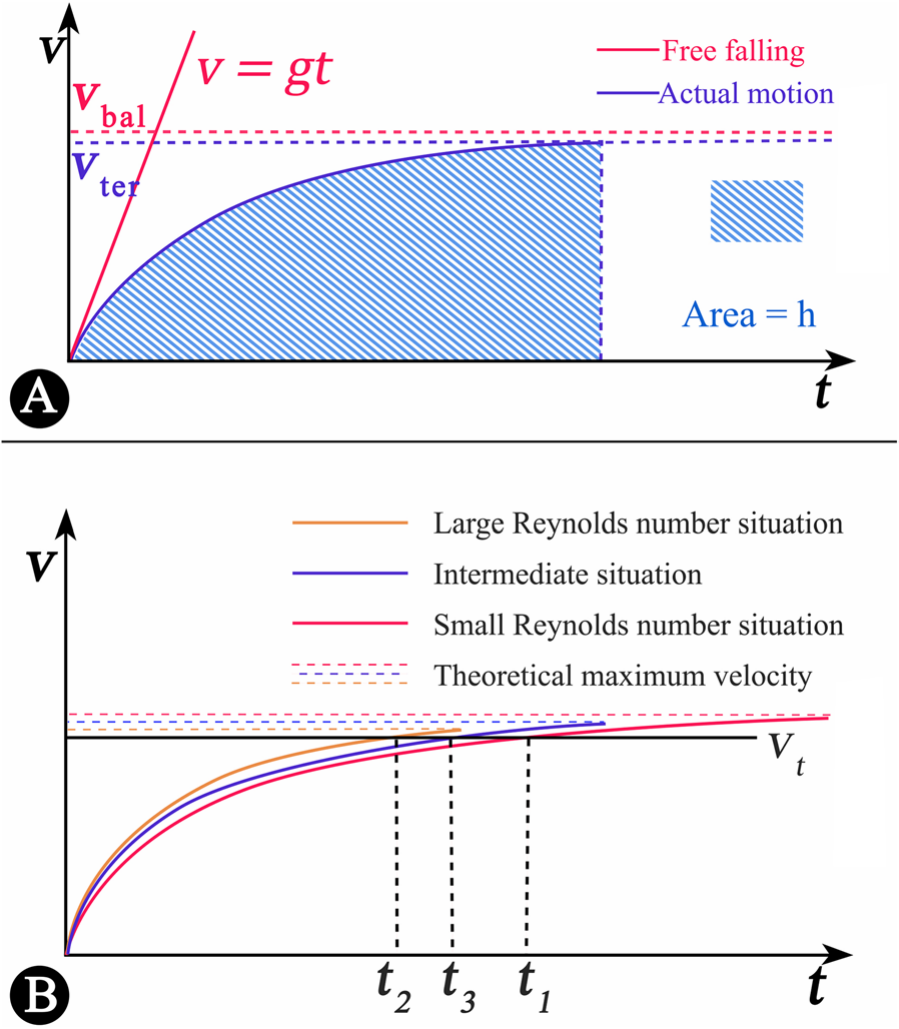
The schematic diagram about the dispersal of the sporophyll units. **A** The velocity-time curve of the falling model. The falling object accelerates air around downward, producing air resistance which opposes gravity. When the object reaches the ground, the velocity of object also reaches the maximum named terminal velocity $\left({v}_{\mathrm{ter}}\right)$. The shaded area is equal to the falling height. If the falling distance large enough, the terminal velocity will converge towards the limit velocity named balanced velocity $\left({v}_{\mathrm{bal}}\right)$. Red solid line shows the case of free fall as a contrast. **B** The approximate velocity–time curve of falling model in three situations. The points ${t}_{1}, {t}_{2},{t}_{3}$ on the horizontal axis represent the time when the falling distance reach 9.0 m in small Reynolds numbers, large Reynolds numbers and intermediate situation.

The dispersal of sporophylls and samaras can be understood as a superposition of the vertical falling and the horizontal motion driven by wind. As the falling velocity increases, upward vertical aerodynamic force gets stronger and finally balances with the weight of sporophyll units. Then the units fall with uniform velocity named ${v}_{\mathrm{bal}}$ (balanced velocity). In fact, the units usually land at a smaller velocity named ${v}_{\mathrm{ter}}$ (terminal velocity) subject to the falling height (Fig. 7A).The approximate hypothetical dispersal distance is the product of falling time and horizontal wind velocity.

## Discussion

### The comparison of sporophyll units

In the Late Devonian, the reduction of functional megaspores suggests one of the evolutionary trends of Isoёtales sensu lato (most of members are arborescent lycopsids) [6, 8, 10, 11] and four equal megaspores in a sporangium is probably regarded as an evolved trait. It is possible that both *Lepidophylloides changxingensis* and *Lepidophylloides longshanensis* are parts of arborescent lycopsids.

In our collections, complete strobili are absent while intact sporophylls with attached sporangia are abundant. The fossil lycopsids may have the megasporangium-sporophyll units widely disseminated [10, 11]. Then we focus on the sporophyll units in the following calculations.

The Reynolds number helps predict patterns in fluid flow situations [12]. For sporophyll units, the characteristic dimension lies in wing width $d$ (the maximum width of lamina of a sporophyll unit) [13]. The Reynolds number (Re) can be defined as

1 $$\mathrm{Re}=\frac{\rho vd}{\mu },$$

where $v$ is the density of air and $\mu$ is the viscosity of medium (air). The latter two are constants. The Reynolds number is thus proportionate to the product of resultant velocity and the maximum width of lamina. The maximum widths (1.7–3.4 mm) of laminae in “small” sporophyll units are close to those (2.6–4.2 mm) in “large” sporophyll units and those (4–9 mm) in model lepidocarps (Table 1).Based on the Reynolds numbers (2116–3582) given by Habgood [9], we infer the Reynolds numbers of “large” and “small” sporophyll units will mainly fall in ${10}^{3}-{10}^{4}$. The Reynolds numbers of similar order of magnitude mean the flow situations are dynamically identical [12], making the comparison of three kinds of sporophyll units more credible.

**Table 1:** Measurements of model lepidocarps (Habgood, 1998), and “Small” and “Large” sporophyll units (in strobilus or disperse) in this study

	Length of lamina (mm)	Maximum width of lamina (mm)	Pedicel width (mm)	Sporangium length (mm)	Height of Sporangium (mm)	Wing loading (mg/cm^2^)
Model lepidocarps	18–41	4.0–9.0	5.0–10	–	–	52.0–80.6
“Small” sporophylls	12–20	1.7–3.4	3.4 -5.3	2.8–4.6	1.5–1.8	72.4–94.5
“Large” sporophylls	15–26	2.6–4.2	6.9–9.8	6.8–9.4	1.8–2.8	233–349

Examining samaras, Green has demonstrated that the value of angular velocity is small compared to that of terminal velocity [14], and the relationship between the samaras’ wing loading and terminal velocity ${v}_{\mathrm{ter}}$ is

1a $$v_{{{\text{ter}}}} \propto \sqrt {\frac{w}{{A_{w} }}} ,$$

where $w$ is the weight of samaras,

A_W_ is the surface area of the wing,

$w/{A}_{W}$ is defined as the samaras’ wing loading.

As for sporophyll units, we also use terminal velocity as an indicator of dispersal ability. Three sporophyll units have similar geometric shapes. It seems that most of mass is concentrated at the sporangium and the difference of sporangium density might be negligible in the “large”, “small” and model lepidocarps. The “large” sporophyll units have greater volume of sporangia than the “small” sporophyll units, and the laminae of “small” and “large” sporophylls are more delicate than those of model lepidocarps. After the estimate of wind loading (Additional file 1: S2, Table 1), we roughly get the relation of the terminal velocity: model lepidocarps < “small” sporophylls < “large” sporophylls. Thus we can use the falling height and terminal velocity of model lepidocarps to estimate the wind dispersal ability of fossil sporophylls in this study.

### The relation of falling time and height of tree

The quantitative relation between the drag and velocity is divided into three cases depending on the Reynolds number (small, large and intermediate Reynolds number situation). The detailed calculation process about wind dispersal is in Additional file (Additional file 1: S3).

The basal equation is.

2 $$mg - D = m\frac{{{\text{d}}v}}{{{\text{d}}t}}$$

For the small Reynolds number situation, there is a linear relationship between the drag $D$ and the velocity $v$ [12]. The equation is:

2a $$\frac{dv}{{{\text{dt}}}} = g - k_{1} v.$$

where ${k}_{1}$ is a constant.

We use y to represent the falling distance in the vertical direction, and we get the equation by eliminating:

2b $${\text{y}} = \frac{1}{{k_{1}^{2} }}\left( {g\ln \left( {\frac{g}{{g - vk_{1} }}} \right) - vk_{1} } \right).$$

Based on the former experiments’ data [9], we put the values into this equation$\nu_{{{\text{ter}}}} = 5.667{\text{m}}/{\text{s}}$, ${y}_{\mathrm{ter}}=9.000 \mathrm{m}$, $g=9.800\mathrm{ m}/{\mathrm{s}}^{2}$). The landing time is named ${t}_{1}$, and the main results can be expressed as:

2c $$\left\{\begin{array}{c}{t}_{1}=2.117 s\\ {v}_{\mathrm{bal}}=5.833 m/s\\ {v}_{\mathrm{ter}}/{v}_{\mathrm{bal}} =0.9715\end{array}\right.$$

For the large Reynolds number situation, the drag is proportional to the square of velocity [12]. The Eq. (2a) is

2d $$\frac{dv}{{dt}} = g - k_{2} v^{2} .$$

($${k}_{2}$$ is a constant.)

Using y to represent the falling distance in the vertical direction, we get

2e $$y=\frac{1}{2{k}_{2}}\mathrm{ln}\left(\frac{g}{g-{k}_{2}{v}^{2}}\right)$$

Plug $\nu_{{{\text{ter}}}} = 5.667{\text{m/s}}$ and ${y}_{\mathrm{ter}}=9.000 \mathrm{m}$ into the equation, we get ${k}_{2}=0.3039 {\mathrm{m}}^{-1}$ (the approximate solution). In the large Reynolds number situation, we use ${t}_{2}$ to represent the landing time. Similarly, we get

2f $$\left\{\begin{array}{c}{t}_{2}=1.973 s\\ {v}_{\mathrm{bal}}=5.679 m/s\\ {v}_{\mathrm{ter}}/{v}_{\mathrm{bal}} =0.9978\end{array}\right.$$

In the intermediate situation, there are hybrid relations between the drag and the velocity:

$$D = A + Bv + Cv^{2} .$$

where A, B, and C are constants [12].

The calculation will be much more complex since the numerical values of A, B, and C were unknown. What seems certain is that the balance velocity in the intermediate situation will fall in between the balance velocity in small and large Reynolds numbers and the shapes of the velocity–time curves are similar (Fig. 7B). Using the curves of these three functions (Fig. 7B), we infer that the falling time ${t}_{3}$ of the intermediate situation (${y}_{\mathrm{ter}}=9.000 \mathrm{m}$) will also fall in between this in small and large Reynolds numbers.

In every situation, the terminal velocity is very close to the balance velocity. If the sporophyll units continue to fall, they will travel at a constant speed in the vertical direction. In summary, when the falling height ${y}_{0}$ is over $9.000\mathrm{ m}$, there is a cap for the falling time ${t}_{0}$:

2g $$t_{0} < \max \left( {t_{1} ,t_{2} ,t_{3} } \right) + \left( {y_{0} - y_{{{\text{ter}}}} } \right)/v_{{{\text{ter}}}} = 2.117 + \left( {y_{0} - 9.000} \right)/5.667$$

Thus the Eq. (2g) represents a practical upper limit of time for the falling model at a predetermined height.

### Wind dispersal and comparison with modern samara

Generally, the potential range of sporophyll dispersal is collectively determined by the wind conditions, growth form or height of arborescent lycopsids, and parameters of sporophylls. The max height of trees is related to the species and geological time [15–17], while the wind conditions are determined by habitat and climate. The realities of wind field can be complicated. Greene & Johnson argued that the variation in horizontal and vertical winds in the forest is much greater than the variation in terminal velocity of diaspores [18]. However, the turbulent intensities of wind will not be as large as mentioned when the indicator changed to maximum dispersal.

**Fig. 8:**
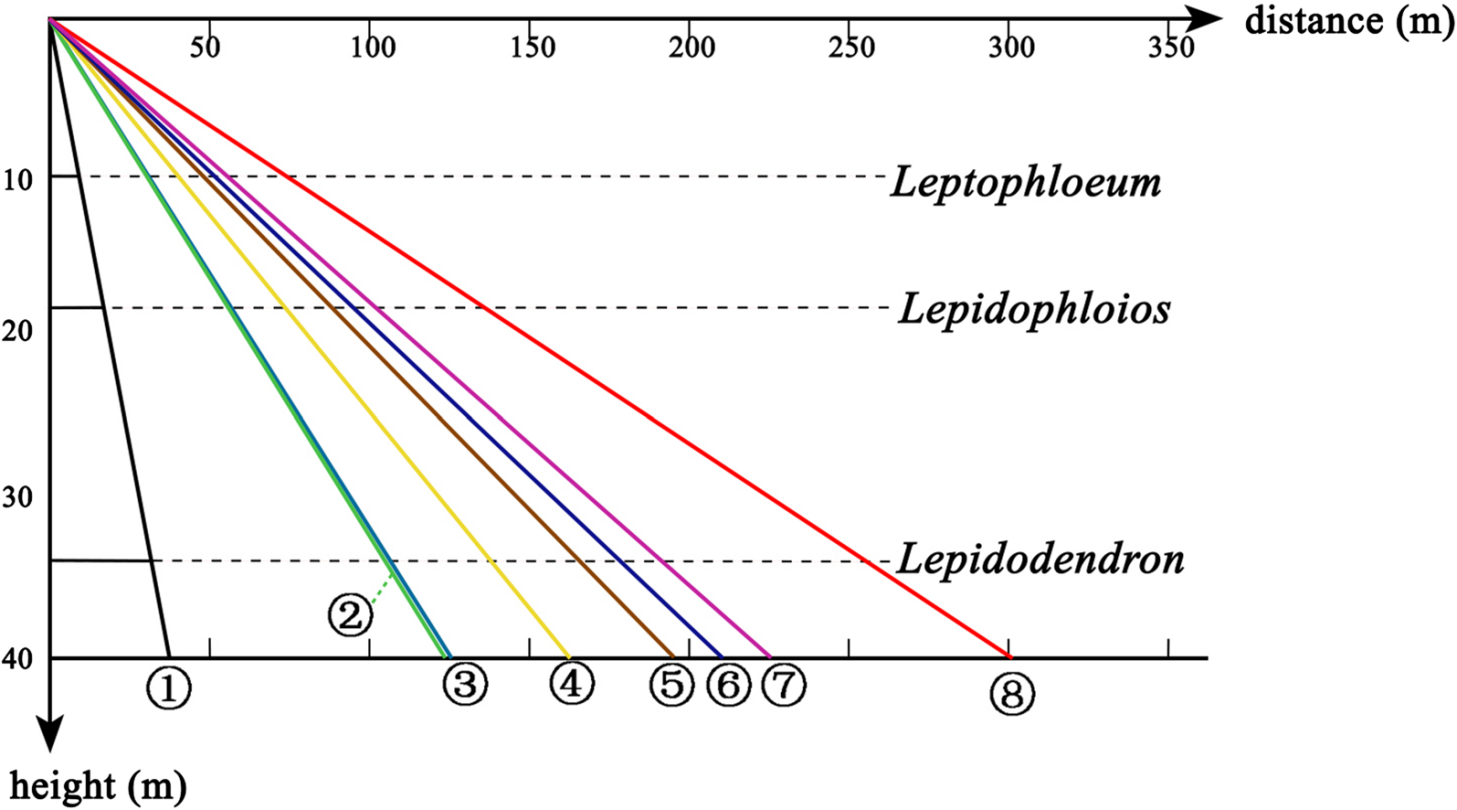
The contrast about potential dispersal ability of the modern spinning samaras and the model lepidocarps with a wind speed of 5 m per second. Data of the modern samaras’ terminal velocity are from Green [15], and we draw lines to show the relationship between height and dispersal distance. Considering the lack of information about the height of the ancient trees, we use the rough height of some Late Paleozoic arborescent lycopsids (*Leptophloeum rhombicum*, *Lepidophloios*, *Lepidodendron*) for reference. 1. The model lepidocarps; 2. Green ash; 3. White ash; 4. Tulip tree; 5. Sugar maple; 6. Box elder; 7. Sliver maple; 8. Red maple.

Using the data of some modern samaras’ terminal velocity (Green, 1980, among 0.660–1.620 m/s), a form is made to describe their acceleration in the falling process (Table 2). It shows all the samaras reach the balanced velocity at very beginning. In other words, the modern samaras and the sporophyll units will travel at a constant speed in the vertical direction after falling a small distance from the start point. Thus the whole falling time can be inferred from the division result of height and terminal velocity of seeds or sporophyll units. Then based on these data of modern samaras, we build a criterion for the dispersal abilities of seeds or sporophyll units from a point source (see in Fig. 8).

**Table 2:** Different Accelerations in the falling progress

Balanced velocity (m/s)	Distance of fall			
	Small Reynolds numbers		Large Reynolds numbers	
	90% (m)	95% (m)	90% (m)	95% (m)
0.500	0.036	0.053	0.021	0.030
1.000	0.143	0.209	0.085	0.119
2.000	0.572	0.835	0.339	0.475
5.679			2.718	3.832
5.833	4.868	7.100		

The distance of fall means when the samaras fall such distance the velocity reaches the appropriate percentage of the balanced velocity

The graph (Fig. 8) shows that the dispersal distance of sporophyll units is far less than that of samaras in the same condition. Compared with the modern samaras, even if the parent plants approach the maximum height (40-50 m) as in the Late Palaeozoic arborescent lycopsids, the spread range of present sporophyll units is rather limited.

During the calculate progress, we leave room for the unexpected variables, and the weak dispersal ability for the sporophyll units is credible.

### Environmental adaptation

In the modern forest, the multiple-layered canopy absorbs the most photosynthetic active radiation. It is significant for the trees to disperse samaras over a sufficient area to ensure that the seedlings can undergo less competition for sunlight and nutrients [19], which is generally intensified due to the narrow ecological space.

However, this is not the case in the Late Devonian. In the turbulent nearshore environment, the slender stems of some tree lycopsids can survive in the strong waves only when they are closely united [3]. Therefore, we propose that the reproductive strategy of some lycopsids is a kind of “slow but steady expansion”, corresponding to the relatively poor samaral behaviour of sporophyll units. In addition, the low canopy closure of the lycopsids leaves enough living space for the saplings. As opportunists or site occupiers, the tree lycopsids could rapidly occupy suitable niches by form dense one-species stands [5]. Therefore the sporophyll units with relatively poor samaral behaviour might be sufficient to meet the demand of dispersal, though further research on their morphology and function is still needed.

## Conclusions

Based on the morphological analysis and the comparison of Reynold numbers, this study demonstrates that the dispersal behaviour of sporophyll units in our materials is comparable to that of the model lepidocarps. The modern samaras and the fossil sporophyll units keep an even vertical velocity after falling a small distance from the start point. Thus the whole falling time can be inferred from the division result of height and terminal velocity of seeds or sporophyll units. The results show the relatively weak dispersal potential of sporophyll units, which may lead to the high density of early forests mainly formed by arborescent lycopsids, as an adaptation to turbulent environment in the Late Devonian.

More studies need to focus on the wind dispersal ability of sporophyll units disseminated by other lycopsids, including the species in both Late Devonian and Permo-Carboniferous. The potential updrafts that enhance the long distance dispersal are not calculated in this study. Further works including simulation experiments and statistical method are needed.

## Materials and methods

### Fossils collection

The fossils were collected from the lower part of the Upper Devonian Wutong (Wutung) Formation at Longshan section (GPS data: 30°51′52''N and 119°44′53''E), Lincheng Town, Changxing County, Zhejiang Province, China (Fig. 1). The age of this formation at Longshan section had been discussed in detail by Wang et al. [20]. The plant-bearing deposit represents the fifth bed of the subdivided strata and consists of gray mudstone. *Latisemenia longshania* and *Archaeopteris* sp. also occur in this bed ([20]; Fig. 2). More than 140 specimens of lycopsids were obtained, including lots of well-preserved reproductive organs. All specimens are deposited in Geological Museum of Peking University, Beijing. Photographs were made with a digital camera and a microscope.

### Measurement and analysis of data

We measure the sporophyll units and perform a multivariate statistical analysis (Multivariate analysis of variance, Principal Component Analysis and UPGMA Clustering) in the software Paleontological Statistics ([21]; PAST, version 3.0), in order to identify possible similarities amongst sporophyll units (The similarity metric used in UPGMA Clustering is Euclidean). Photoshop CC is used to make figures.

### The ideal model of falling

To build our falling model, we conduct kinematic analysis. The dispersal of sporophylls and samaras can be understood as a superposition of the vertical falling and the horizontal motion driven by wind.

During the vertical fall in the still air, sporophyll units accelerate under the action of a gravitational field. Falling object accelerates air around downward, producing air resistance which opposes gravity [14]. Through force analysis of vertical direction, simple equation is developed based on Newton's Second Law, neglecting aerostatic buoyancy:

$$mg - D = m\frac{{{\text{d}}v}}{{{\text{d}}t}}.$$

where the object’s mass is $m$, $g$ is the acceleration due to gravity, $D$ is the aerodynamic drag [12].

The assumed wind condition is a stable horizontal wind field.

Our aim is to find the relationship between the height of tree and the horizontal movement of a diaspore. By the study of vertical fall, we calculate the accurate falling time ${t}_{\mathrm{ter}}$.

We define a function $D(t)$ to represent the hypothetical dispersal distance.

By definition,

$$D\left( t \right) = \int_{0}^{{t_{{{\text{ter}}}} }} {v_{{{\text{hor}}}} dt} .$$

Ideally, the wind is the only driving force in horizontal direction. In this case, the horizontally moving velocity of a diaspore is no more than the horizontal wind velocity from the start to the end. That means,

$$v_{{{\text{hor}}}} < v_{{{\text{win}}}} \forall t \in \left[ 0 \right.\left. {t_{{{\text{ter}}}} } \right]$$

Then $$D\left(t\right)<{v}_{\mathrm{win}}{t}_{\mathrm{ter}}$$.

To simplify this discussion, we consider the product (the time falling in the still air and horizontal wind velocity) as a hypothetical dispersal distance approximatively.

## Supplementary Information

**Additional file 1: Table S1. **Morphological database of the sporophyll units (dispersed) in this study. **S2. **The detailed calculation process about wing loading. **S3. **The detailed calculation process about wind dispersal.

## Data Availability

All data generated or analyzed during this study are included in this published article and its Additional file 1.
