# Supplementary material for: The morphometric of lycopsid sporophylls and the evaluation of their dispersal potential: an example from the Upper Devonian of Zhejiang Province, China
Source: BMC Ecol Evol. 2021 Nov 3;21:198. doi: 10.1186/s12862-021-01933-3 (PMC8565055; doi:10.1186/s12862-021-01933-3)
Supplement: Supplementary file 1 — Additional file 1: Table S1. Morphological database of the sporophyll units (dispersed) in this study. S2. The detailed calculation process about wing loading. S3. The detailed calculation process about wind dispersal. [file 12862_2021_1933_MOESM1_ESM.docx]

**Table S1. Morphological database of the sporophyll units (dispersed) in this study**

| **Serial number** | **Type** | **Lp (mm)** | **Th**  **(mm)** | **Ps**  **(mm)** | **Ds**  **(mm)** | **Lh**  **(mm)** | **Th /Lp** | **Ps /Lp** | **Ds /Lp** | **Lh /Lp** |
| --- | --- | --- | --- | --- | --- | --- | --- | --- | --- | --- |
| **1** | S | 4.25 | 1.80 | 1.59 | 0.88 | 0.94 | 0.424 | 0.374 | 0.207 | 0.221 |
| **2** | L | 6.58 | 2.13 | 0.53 | 0.16 | 1.31 | 0.324 | 0.081 | 0.024 | 0.199 |
| **3** | S | 4.05 | 1.78 | 1.63 | 0.77 | 1.11 | 0.440 | 0.402 | 0.190 | 0.274 |
| **4** | S | 4.52 | 1.81 | 1.96 | 1.12 | 1.37 | 0.400 | 0.434 | 0.248 | 0.303 |
| **5** | L | 7.77 | 2.73 | 2.16 | 0.49 | 1.56 | 0.351 | 0.278 | 0.063 | 0.201 |
| **6** | L | 7.00 | 2.58 | 2.10 | 0.39 | 1.22 | 0.369 | 0.300 | 0.056 | 0.174 |
| **7** | L | 8.44 | 2.63 | 2.70 | 0.89 | 1.65 | 0.312 | 0.320 | 0.105 | 0.195 |
| **8** | L | 7.92 | 2.22 | 1.62 | 0.43 | 1.10 | 0.280 | 0.205 | 0.054 | 0.139 |
| **9** | S | 4.24 | 1.51 | 1.66 | 0.78 | 1.09 | 0.356 | 0.392 | 0.184 | 0.257 |
| **10** | L | 8.18 | 2.64 | 1.64 | 0.16 | 1.25 | 0.323 | 0.200 | 0.020 | 0.153 |
| **11** | L | 7.22 | 2.34 | 1.37 | 0.39 | 1.30 | 0.324 | 0.190 | 0.054 | 0.180 |
| **12** | L | 7.96 | 2.68 | 1.45 | 0.35 | 0.98 | 0.337 | 0.182 | 0.044 | 0.123 |
| **13** | L | 8.47 | 2.68 | 1.34 | 0.22 | 1.36 | 0.316 | 0.158 | 0.026 | 0.161 |
| **14** | L | 8.39 | 2.16 | 2.59 | 0.54 | 1.33 | 0.257 | 0.309 | 0.064 | 0.159 |
| **15** | S | 4.28 | 1.58 | 1.82 | 1.14 | 1.11 | 0.369 | 0.425 | 0.266 | 0.259 |
| **16** | L | 8.76 | 2.07 | 2.00 | 0.62 | 0.94 | 0.236 | 0.228 | 0.071 | 0.107 |
| **17** | L | 6.02 | 2.11 | 0.55 | 0.08 | 1.02 | 0.350 | 0.091 | 0.013 | 0.169 |
| **18** | L | 6.63 | 2.76 | 0.69 | 0.39 | 1.13 | 0.416 | 0.104 | 0.059 | 0.170 |
| **19** | S | 4.35 | 1.52 | 1.63 | 1.29 | 1.28 | 0.349 | 0.375 | 0.297 | 0.294 |
| **20** | L | 7.33 | 2.27 | 0.73 | 0.23 | 1.32 | 0.310 | 0.100 | 0.031 | 0.180 |
| **21** | L | 7.51 | 2.25 | 1.72 | 0.49 | 0.95 | 0.300 | 0.229 | 0.065 | 0.126 |
| **22** | L | 8.63 | 2.61 | 1.28 | 0.41 | 1.00 | 0.302 | 0.148 | 0.048 | 0.116 |
| **23** | L | 8.19 | 2.27 | 1.32 | 0.43 | 1.09 | 0.277 | 0.161 | 0.053 | 0.133 |
| **24** | L | 9.31 | 2.44 | 2.73 | 0.82 | 1.43 | 0.262 | 0.293 | 0.088 | 0.154 |
| **25** | L | 7.79 | 2.23 | 1.39 | 0.48 | 0.48 | 0.286 | 0.178 | 0.062 | 0.062 |
| **26** | L | 8.51 | 2.53 | 2.62 | 0.70 | 1.14 | 0.297 | 0.308 | 0.082 | 0.134 |
| **27** | L | 8.29 | 2.30 | 1.93 | 0.47 | 1.37 | 0.277 | 0.233 | 0.057 | 0.165 |
| **28** | S | 3.39 | 1.46 | 1.33 | 1.27 | 1.07 | 0.431 | 0.392 | 0.375 | 0.316 |
| **29** | S | 4.30 | 1.62 | 1.45 | 1.06 | 1.26 | 0.377 | 0.337 | 0.247 | 0.293 |
| **30** | L | 8.52 | 2.15 | 1.84 | 0.33 | 0.97 | 0.252 | 0.216 | 0.039 | 0.114 |
| **31** | L | 8.96 | 2.78 | 2.70 | 0.74 | 1.20 | 0.310 | 0.301 | 0.083 | 0.134 |
| **32** | L | 8.35 | 2.15 | 2.90 | 0.63 | 1.26 | 0.257 | 0.347 | 0.075 | 0.151 |
| **33** | L | 9.80 | 2.86 | 2.88 | 0.27 | 1.76 | 0.292 | 0.294 | 0.028 | 0.180 |
| **34** | L | 9.18 | 2.57 | 1.18 | 0.75 | 1.58 | 0.280 | 0.129 | 0.082 | 0.172 |
| **35** | L | 8.32 | 2.36 | 2.59 | 0.59 | 1.55 | 0.284 | 0.311 | 0.071 | 0.186 |
| **36** | L | 7.98 | 2.68 | 2.54 | 0.39 | 1.24 | 0.336 | 0.318 | 0.049 | 0.155 |
| **37** | L | 7.30 | 2.49 | 0.74 | 0.20 | 1.19 | 0.341 | 0.101 | 0.027 | 0.163 |
| **38** | S | 3.74 | 1.65 | 1.27 | 0.93 | 1.20 | 0.441 | 0.340 | 0.249 | 0.321 |
| **39** | S | 3.73 | 1.62 | 1.49 | 1.16 | 0.94 | 0.434 | 0.399 | 0.311 | 0.252 |
| **40** | L | 9.24 | 2.66 | 1.63 | 0.54 | 1.80 | 0.288 | 0.176 | 0.058 | 0.195 |
| **41** | S | 4.12 | 1.70 | 1.75 | 0.92 | 1.37 | 0.413 | 0.425 | 0.223 | 0.333 |
| **42** | L | 8.13 | 2.64 | 1.63 | 0.75 | 1.94 | 0.325 | 0.200 | 0.092 | 0.239 |
| **43** | L | 8.83 | 2.62 | 2.60 | 0.85 | 1.30 | 0.297 | 0.294 | 0.096 | 0.147 |
| **44** | L | 9.58 | 2.22 | 1.15 | 0.30 | 1.96 | 0.232 | 0.120 | 0.031 | 0.205 |
| **45** | L | 8.23 | 2.17 | 2.66 | 0.72 | 1.44 | 0.264 | 0.323 | 0.087 | 0.175 |
| **46** | L | 7.57 | 2.21 | 2.39 | 0.39 | 1.75 | 0.292 | 0.316 | 0.052 | 0.231 |
| **47** | L | 7.75 | 2.32 | 0.00 | 0.00 | 1.16 | 0.299 | 0.000 | 0.000 | 0.150 |
| **48** | L | 8.08 | 2.42 | 2.05 | 0.73 | 1.52 | 0.300 | 0.254 | 0.090 | 0.188 |
| **49** | L | 8.17 | 2.70 | 1.90 | 0.88 | 1.59 | 0.330 | 0.233 | 0.108 | 0.195 |
| **50** | L | 8.10 | 2.17 | 1.48 | 0.21 | 1.17 | 0.268 | 0.183 | 0.026 | 0.144 |
| **51** | S | 4.23 | 1.50 | 1.41 | 1.02 | 1.04 | 0.355 | 0.333 | 0.241 | 0.246 |
| **52** | L | 8.69 | 2.56 | 2.10 | 0.98 | 1.79 | 0.295 | 0.242 | 0.113 | 0.206 |
| **53** | L | 8.77 | 2.56 | 0.74 | 0.40 | 1.22 | 0.292 | 0.084 | 0.046 | 0.139 |
| **54** | L | 8.31 | 2.36 | 1.29 | 0.83 | 1.28 | 0.284 | 0.155 | 0.100 | 0.154 |
| **55** | L | 8.75 | 2.24 | 1.56 | 0.72 | 1.17 | 0.256 | 0.178 | 0.082 | 0.134 |
| **56** | L | 8.43 | 2.65 | 3.01 | 0.99 | 1.62 | 0.314 | 0.357 | 0.117 | 0.192 |
| **57** | S | 4.82 | 1.63 | 1.66 | 1.00 | 1.50 | 0.338 | 0.344 | 0.207 | 0.311 |
| **58** | L | 9.13 | 2.36 | 1.98 | 0.94 | 1.59 | 0.258 | 0.217 | 0.103 | 0.174 |
| **59** | S | 4.58 | 1.78 | 1.45 | 0.82 | 1.46 | 0.389 | 0.317 | 0.179 | 0.319 |
| **60** | L | 7.49 | 2.43 | 1.47 | 0.55 | 1.34 | 0.324 | 0.196 | 0.073 | 0.179 |
| **61** | L | 7.80 | 2.70 | 0.59 | 0.34 | 1.53 | 0.346 | 0.076 | 0.044 | 0.196 |
| **62** | L | 8.18 | 2.78 | 1.38 | 0.85 | 1.46 | 0.340 | 0.169 | 0.104 | 0.178 |

Lp: length of pedicel; Th: thickness; Lh: length of heel; Ps: position of separation; Ds: distance of separation. Type S: “small” sporophyll units; Type L:“large” sporophyll units.

**S2. The detailed calculation process about wing loading**

**Precondition:**

Definition： $WL=w/A_{W}$.

Where $WL$ is the wing loading, $w$is the weight of samaras, *A_W_* is the surface area of the wing.

Most of mass is concentrated at the sporangium:

$w_{sporangium}=\left( 0.8-0.9 \right)\times w_{sporophyll units}$,

$w_{sporophyll units}=(1.11-1.25)w_{sporangium}$.

$w_{sporangium}=\rho V$.

Where $\rho$ and $V$ are the density and volume of sporangium.

The shape of a sporangium is like an ellipsoid, and the expression of volume formula is

$V=\frac{4\pi}{3}abc$.

Where a, b, c are length, width and height of the sporangium. Most sporophyll units preserved in lateral and abaxial view, makes the observation of sporangium difficult. So we use the width of pedicel as the width of sporangium since most of the sporangia are completely covered by pedicels in the abaxial view.

Habgood (1998) find mung beans are of both the correct size and density to simulate the sporangia of lepidocarps. It seems that most of mass is concentrated at the sporangium and the difference of sporangium density might be negligible in the “large”, “small” and model lepidocarps. And we take $1.35 g/\mathrm{cm}^{3}$ (the average density of main beans) as the density $\rho$ in the calculation.

The laminae of sporophyll units are correspond to the wings of samaras, and the laminae is triangular in shape. Then the expression of area formula is:

$A_{W}=\frac{1}{2}w_{lamina}l_{lamina}$.

Where the $w_{lamina}$ is the max width of lamina.

**Results:**

The wing loading of lepidocarps: $52.0-80.6 mg/\mathrm{cm}^{2}$ (data from the Table 3, the square of the last columns, Habgood, 1998)

The wing loading of “small” sporophyll units: $72.4-94.5 mg/\mathrm{cm}^{2}$

The wing loading of “large” sporophyll units: $233-324 mg/\mathrm{cm}^{2}$

**References:**

Habgood, K.S., Hemsley, A.R. & Thomas, B.A. 1998. Modelling of the dispersal of Lepidocarpon based on experiments using reconstructions. Review of Palaeobotany & Palynology, 102(1-2), 101-114.

**S3. The detailed calculation process about wind dispersal**

The basal equation is

$mg-D=m\frac{ⅆv}{ⅆt}$. (2-1)

For the small Reynolds number situation, since the drag $D$ is linearly related to the velocity $v$ ($D=mk_{1}v$), we get

$\frac{dv}{\mathrm{dt}}=g-k_{1}v$. (2-2)

We create the function relation between velocity $v$ and time $t$ ($v=v\left( t \right)$), and the relation of falling distance $y$ and time $t$ ($y=y\left( t \right))$, then

$v^{'}\left( t \right)=g-k_{1}v\left( t \right)$.

We define a new function $w\left( t \right)=\frac{g}{k_{1}}-v\left( t \right)$, then

$$v\left( t \right)=\frac{g}{k_{1}}-w\left( t \right)$$

$$w’\left( t \right)=-v^{'}\left( t \right)=k_{1}v\left( t \right)-g=-k_{1}w\left( t \right)$$

$\frac{w'\left( t \right)}{w\left( t \right)}=-k_{1}$.$\left( w\left( t \right)>0 \right)$

Let’s integrate both sides,

$\ln\left| w\left( t \right) \right|=-k_{1}t+C1$ ($C1$ is a constant)

$$w\left( t \right)=e^{-k_{1}t+C1}=e^{-k_{1}t}*e^{C1}$$

$v\left( t \right)=\frac{g}{k_{1}}-w\left( t \right)=\frac{g}{k_{1}}-e^{-k_{1}t}*e^{C1}$.

When $t=0$, $v\left( t \right)=0$, then $\frac{g}{k_{1}}-e^{C1}=0$

$v\left( t \right)=\frac{g}{k_{1}}-e^{-k_{1}t}\frac{g}{k_{1}}=\frac{g}{k_{1}}\left( 1-e^{-k_{1}t} \right)$.

The equation also can be written as

$v=\frac{g}{k_{1}}\left( 1-e^{-k_{1}t} \right)$

$$t=-\frac{\ln\left( 1-\frac{k_{1}v}{g} \right)}{k_{1}}$$

$y=y\left( t \right)=\int v\left( t \right)dt=\frac{g}{k_{1}}\left[ t-\frac{1}{k_{1}}(1-e^{-k_{1}t}) \right]$

Based on definition of Physics, $\int v\left( t \right)d_{t}=y\left( t \right),$ then

$$\int\left( \frac{dv}{dt} \right)d_{t}=\int\left( g-k_{1}v\left( t \right) \right)d_{t}$$

$$v\left( t \right)=\int gd_{t}-\int k_{1}v\left( t \right)d_{t}$$

$$v\left( t \right)=gt-k_{1}y\left( t \right)$$

$$y\left( t \right)=\frac{g}{k_{1}}(t-\frac{v\left( t \right)}{g})$$

$y=\frac{g}{k_{1}}(t-\frac{v}{g})$.

Eliminating the variable $t$, then

$$y{k_{1}}^{2}+vk_{1}=k_{1}gt=-g\ln\left( 1-\frac{k_{1}v}{g} \right)=g\ln\left( \frac{g}{g-vk_{1}} \right)$$

$y=\frac{1}{{k_{1}}^{2}}\left( g\ln\left( \frac{g}{g-vk_{1}} \right)-vk_{1} \right)$. (2-3)

Based on the former experiments’ data [9], we put the values into this equation ($\nu_{\mathrm{ter}}=5.667 m/s$, $y_{\mathrm{ter}}=9.000 m$, $g=9.800 m/s^{2}$). The landing time is named $t_{1}$ to distinguish from the other situation. The equation is solved approximatively by the graph of functions, and the solution (using international unit system) is:

$\left\{ \begin{aligned} k_{1}=1.680 \\ t_{1}=2.117 \end{aligned} \right.$.

When $v=v_{\mathrm{bal}}$, $g-k_{1}v=0$, then

$k_{1}=\frac{g}{v_{\mathrm{bal}}}$.

$In the limit t\to\infty$, $v\to\frac{g}{k_{1}}=v_{\mathrm{bal}}$, $v_{\mathrm{bal}}=5.833 m/s$, $v_{\mathrm{ter}}/v_{\mathrm{bal}} =0.9715$.

And the main results can be expressed as：

$\left\{ \begin{aligned} t_{1}=2.117 s \\ v_{\mathrm{bal}}=5.833 m/s \\ v_{\mathrm{ter}}/v_{\mathrm{bal}} =0.9715 \end{aligned} \right.$ (2-4)

For the large Reynolds number situation, the drag is proportional to the square of velocity. The equation (2-1) is

$\frac{dv}{\mathrm{dt}}=g-k_{2}v^{2}$. (2-5)

$$dt=\frac{dv}{g-k_{2}v^{2}}$$

Let $\frac{dv}{\mathrm{dt}}=0$, $v=v_{bal}=\sqrt{\frac{g}{k_{2}}}$.

$y=\int vdt=\int\frac{vdv}{g-k_{2}v^{2}}=\frac{1}{2}\int\frac{dv^{2}}{g-k_{2}v^{2}}=-\frac{1}{2k_{2}}\ln\left( \frac{g}{k_{2}}-v^{2} \right)+C2$.

($C2$ is a constant)

When $t=0$, $v=0$and $y=0$, then

$0=-\frac{1}{2k_{2}}\ln\left( \frac{g}{k_{2}} \right)+C$2

$C2=\frac{1}{2k_{2}}\ln\left( \frac{g}{k_{2}} \right)$,

$y=-\frac{1}{2k_{2}}\ln\left( \frac{g}{k_{2}}-v^{2} \right)+\frac{1}{2k_{2}}\ln\left( \frac{g}{k_{2}} \right)=\frac{1}{2k_{2}}\ln\left( \frac{g}{g-k_{2}v^{2}} \right)$, (2-6)

Plug $\nu_{\mathrm{ter}}=5.667 m/s$ and $y_{\mathrm{ter}}=9.000 m$ into the equation, we get $k_{2}=0.3039 m^{-1}$ (the approximate solution).

For $v\in\left[ 0 \right.,\left. \sqrt{\frac{g}{k_{2}}} \right)$, using variable substitution,

$\nu=\sqrt{\frac{g}{k_{2}}}\sin\alpha,$ $(\alpha\in\left[ 0 \right.,\frac{\pi}{2}))$.

And if $t=0$, then $v=0$, $\sin\alpha=0$, $\alpha=0$

Then

$y=\frac{1}{2k_{2}}\ln\left( \frac{g}{g-k_{2}v^{2}} \right)=\frac{1}{2k_{2}}\ln\left( \frac{1}{1-\left( \sin\alpha\right)^{2}} \right)=\frac{1}{2k_{2}}\ln\left( \frac{1}{\left( \cos\alpha\right)^{2}} \right)=\frac{1}{2k_{2}}\left[ 2\left( \ln\frac{1}{\cos\alpha} \right) \right]$

$y=\frac{1}{k_{2}}\ln\frac{1}{\cos\alpha}$,

Plug $y_{\mathrm{ter}}=9.000 m$ into the equation, we get $\cos\alpha=0.06630$.

Then $\sin\alpha={v_{\mathrm{ter}}}/{v_{\mathrm{bal}}}=0.9978$

$v_{\mathrm{bal}}=\frac{\nu_{\mathrm{ter}}}{\sin\alpha}=5.679 m/s$.

Based on equation (2-5),

$dt=\frac{dv}{g-k_{2}v^{2}}$

$t=\int\frac{dv}{g-k_{2}v^{2}}$

$t=\int\frac{\sqrt{\frac{g}{k_{2}}}d\sin\alpha}{g-k_{2}\frac{g}{k_{2}}\left( \sin\alpha\right)^{2}}$

$t=\frac{1}{\sqrt{k_{2}g}}\int\frac{\cos\alpha d\alpha}{\left( \cos\alpha\right)^{2}}$

$t=\frac{1}{\sqrt{k_{2}g}}\int\left( \frac{1}{\cos\alpha} \right)d\alpha$

$t=\frac{1}{\sqrt{k_{2}g}}\int\left( \sec\alpha\right)d\alpha$

$t=\frac{1}{\sqrt{k_{2}g}}\left[ \ln\left| \sec\alpha+\tan\alpha\right| \right]+C3$.

When $t=0$, then $\alpha=0$, $\sin\alpha=0$, $\cos\alpha=1$, $\sec\alpha=1$, $\tan\alpha=0$,

$0=\frac{1}{\sqrt{k_{2}g}}\ln\left( 1+0 \right)+C3$,

$$C3=0$$

$t=\sqrt{\frac{1}{k_{2}g}}\ln\left| \sec\alpha+\tan\alpha\right|$,

$t=\frac{1}{\sqrt{k_{2}g}}\ln\frac{1+\sin\alpha}{\cos\alpha}$.

Plug the value of $g, k_{2},\sin\alpha,\cos a$, we get $t_{2}=1.973 s$.

Similarly, the main results can be expressed as:

$\left\{ \begin{aligned} t_{2}=1.973 s \\ v_{bal}=5.679 m/s \\ v_{t}/v_{bal} =0.9978 \end{aligned} \right.$ (2-7)

In small Reynolds number situation, we call a moment $t_{0}$($t_{0}>t_{1})$, the corresponding distance $y_{0}$($y_{0}>9.000 m)$, and the velocity $v_{0}$. For any $t_{0}>t_{1}$, we have $v_{0}\in\left( v_{t},v_{\mathrm{bal}} \right)$.

Then $v_{\mathrm{bal}}(t_{0}-t_{1}$)$>y_{0}-y_{t}>v_{t}(t_{0}-t_{1}$),

$t_{0}<t_{1}+\left( y_{0}-y_{t} \right)/{v_{t}}$.

Similarly, in the large Reynolds number situation and intermediate situation, we get

$t_{0}<t_{2}+\left( y_{0}-y_{1} \right)/{v_{t}}$;

$t_{0}<t_{3}+\left( y_{0}-y_{1} \right)/{v_{t}}$

Then in summary,

$t_{0}<\max\left( t_{1},t_{2},t_{3} \right)+\left( y_{0}-y_{t} \right)/{v_{t}}=2.117+\left( y_{0}-9.000 \right)/{5.667}$. (2-8)
